# An ortho­rhom­bic polymorph of 2-(1,3,5-di­thia­zinan-5-yl)ethanol or MEA-di­thia­zine

**DOI:** 10.1107/S2056989022000342

**Published:** 2022-01-14

**Authors:** Nate Schultheiss, Jeremy Holtsclaw, Matthias Zeller

**Affiliations:** a Davidson School of Chemical Engineering, Purdue University, 480 W. Stadium Ave., West Lafayette, IN 47907, USA; b Pioneer Oil, 400 Main Street, Vincennes, IN, 47951, USA; c Purdue University, Department of Chemistry, 560 Oval Dr., West Lafayette, IN 47907, USA

**Keywords:** polymorphism, di­thia­zine, hydrogen bonding, hydrogen sulfide, crystal structure

## Abstract

Substituted triazines are a class of compounds utilized for scavenging and sequestering hydrogen sulfide in oil and gas production operations. The reaction of one of these triazines under field conditions resulted in the formation of the title compound, 2-(1,3,5-di­thia­zinan-5-yl)ethanol, C_5_H_11_NOS_2_, or MEA-di­thia­zine. Polymorphic form I, in space group *I*4_1_/*a*, was first reported in 2004 and its extended structure displays one-dimensional, helical strands connected through O—H⋯O hydrogen bonds. We describe here the form II polymorph of the title compound, which crystallizes in the ortho­rhom­bic space group *Pbca* as centrosymmetric dimers through pairwise O—H⋯N hydrogen bonds from the hydroxyl moiety to the nitro­gen atom of an adjacent mol­ecule.

## Abstract 2

The use of the H_2_S scavenger mono­ethano­lamine triazine in a natural gas treatment facility produced a new *Pbca* polymorph (form II) of 2-(1,3,5-di­thia­zinan-5-yl)ethanol featuring O—H⋯N hydrogen-bonded dimers rather than one-dimensional, helical O—H⋯O strands as reported previously for the *I*4_1_/*a* (form I) polymorph.

## Chemical context

Hydrogen sulfide is a corrosive and lethal gas that is commonly encountered during the production of hydro­carbons from subterranean reservoirs (Marriott *et al.*, 2016 ▸). The highly toxic H_2_S gas must be removed from the hydro­carbon stream to ensure a commercially salable product into the pipeline and refinery distribution network (Kermani *et al.*, 2006 ▸). One of the most widely applied economical and effective strategies is to scavenge the hydrogen sulfide through the use of scavenger chemicals such as hexa­hydro-1,3,5-tris­(hy­droxy­eth­yl)-*s*-triazine, colloquially referred to as mono­ethano­lamine triazine (MEA-triazine) (Taylor *et al.*, 2017 ▸). The MEA-triazine is routinely administered at remote field locations using engineering controls that enhance the efficiency with which the hydrogen sulfide reacts with the MEA-triazine scavenger. 
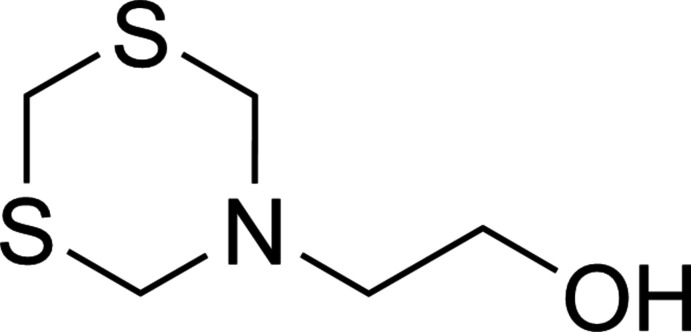


**Figure 1:**
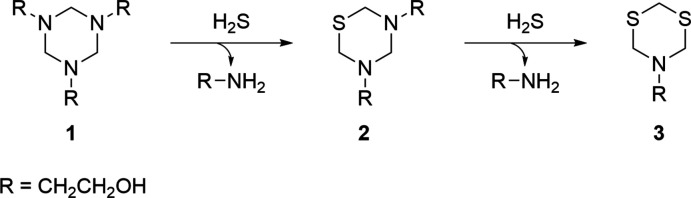
Reaction of hydrogen sulfide with 2-(1,3,5-di­thia­zinan-5-yl)ethanol (MEA-triazine) (**1**) to yield the MEA mono- (**2**) and di­thia­zine (**3**) products.

The reaction of hydrogen sulfide with the MEA-triazine (**1**) scavenger has been described, see Fig. 1 ▸ (Bakke *et al.*, 2001 ▸; Wylde *et al.*, 2020 ▸). Furthermore, while hydrogen sulfide can theoretically react with a third mol­ecule to yield a tri­thiane mol­ecule, this reaction does not proceed under most conditions. As a result, sparingly soluble solid di­thia­zine (**3**) mol­ecules or an amorphous polymerized di­thia­zine material are the typical final products under aqueous reaction conditions (Taylor & Matherly, 2011 ▸; Taylor *et al.*, 2013 ▸; Wang *et al.*, 2020 ▸). Engineering protocols are implemented into the pipeline flow systems so that the sparingly soluble solids from the spent scavenger are continuously removed. However, these operations are occasionally not completely effective in eliminating solids and a buildup of intra­ctable residues in the system will result, thus impeding the flow path and complicating field operations. This was the case at one production facility, where the title compound (**3**) crystallized within the field treatment system.

The mol­ecular and crystal structure of title compound **3** has been determined before. The first polymorph, form I, in space group *I*4_1_/*a* was described independently by Galvez-Ruiz *et al.* (2004 ▸) and Wang *et al.* (2020 ▸). Recently, a second polymorph, in space group *Pbca*, was disclosed as a CSD communication (Unruh, 2021 ▸). No further details, such as the origin of the material, the solvent or mode of crystallization or other information was stated with the deposited cif file and the mol­ecular and crystal structures were not discussed.

## Structural commentary

Single crystals formed in the field treatment system and were used for intensity data collection without recrystallization. Data were collected at both room temperature as well as 150 K. The polymorph obtained was that of form II, in space group *Pbca*, and the data are in good agreement with those recently disclosed by Unruh (2021 ▸).

Phase purity of the sample as harvested from the field treatment system was verified by powder X-ray diffraction analysis (supplementary Figures S1 and S2). No phase change was observed between 150 K and room temperature. The unit-cell parameters obtained by Rietveld analysis of the structural model against the room-temperature powder pattern are *a* = 9.6596 (6), *b* = 8.9248 (5), *c* = 17.748 (1) Å and *V* = 1530.03 (15) Å^3^ (Table S1A), matching the parameters from the room-temperature single-crystal data collection (Table 4 ▸). Powder data and unit-cell parameters from samples recrystallized from either iso­propanol or an iso­propanol–water mixture are indistinguishable from those of the field sample (Fig. S1, Fig. S2, Table S1).

**Figure 2:**
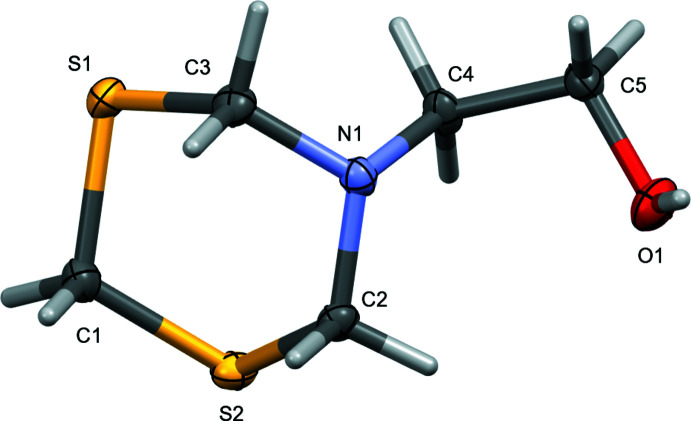
The mol­ecular structure of 2-(1,3,5-di­thia­zinan-5-yl)ethanol. Displacement ellipsoids are shown at the 50% probability level.

The mol­ecular conformations and packing at low temperature and room temperature are essentially identical and unless otherwise noted, the following discussion will be based on the 150 K data set. The title compound (Fig. 2 ▸) crystallizes in the ortho­rhom­bic space group *Pbca*. Bond distances and angles are in the expected ranges and unexceptional. Key torsion angles are given in Table 1 ▸. The nitro­gen atom is *sp* ^3^ hybridized, as expected for a tris­(methyl­ene) substituted amine [C—N—C angles range from 113.16 (5) to 114.49 (5) °, the bond angle sum is 342.06°], the di­thia­zine ring exhibits the expected chair conformation, and the ethano­lamine unit is substituted onto the ring in an axial orientation. The title compound has this in common with the other structures featuring a MEA-di­thia­zine moiety: The previously reported polymorph (form I) in space group *I*4_1_/*a* and also the bromide and chloride salts of MEA-di­thia­zine, which are protonated at the amine and isomorphous (Bushmarinov *et al.*, 2009 ▸; Galvez-Ruiz *et al.*, 2008 ▸), also feature an *sp* ^3^-hybridized amine N atom, as well as a chair conformation di­thia­zine ring axially substituted by the ethano­lamine.

The most flexible fragment of the MEA-di­thia­zine mol­ecule, and worth a closer investigation, is the ethano­lamine chain. In the form II polymorph the N1—C4—C5—O1 chain adopts a *gauche* conformation, with a torsion angle of 65.09 (7)° (Table 2 ▸). In the form I polymorph the conformation is also *gauche*, with a torsion angle of −70.1 (3)° (in the chosen enanti­omer). In the bromide and chloride salts, this torsion angle is slightly smaller (55.1, 54.2 and −55.0° in the three published structures), but also *gauche*. The form I polymorph and the salts thus exhibit the same chair conformation and axial position of the ethano­lamine group as well as a common *gauche* conformation of the ethano­lamine chain as observed for form II of the title compound, indicating a clear preference of the MEA-di­thia­zine mol­ecule towards this specific mol­ecular conformation.

**Figure 3:**
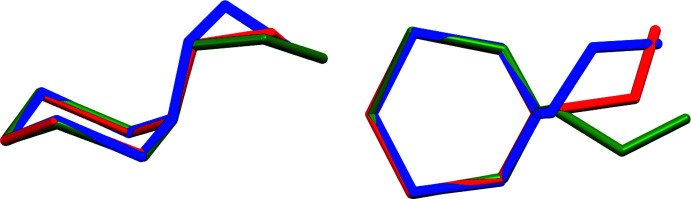
An overlay of the two polymorphs of MEA-di­thia­zine (red: form II; blue form I) and of the cation of the chloride salt (green). Overlays are based on a least-squares fit of the di­thia­zine ring atoms.

What does differ between the three structures involving MEA-di­thia­zine or its cation are the C—N—C—C torsion angles between the di­thia­zine ring and the ethyl group. In form II, they are −104.74 (6)° (C2—N1—C4—C5) and 122.32 (6)° (C3—N1—C4—C5). The equivalent torsion angles in form I are 78.9 (3) and −148.1 (3)°, respectively, and those of the chloride salt are 175.8 and −55.2° (Bushmarinov *et al.*, 2009 ▸). Fig. 3 ▸, an overlay of the mol­ecules from the three crystalline motifs, exemplifies the similarities and differences. The cause for the differences between the two polymorphs and the salt can be found among the inter­molecular and packing inter­actions, to be discussed below.

## Supra­molecular features

**Figure 4:**
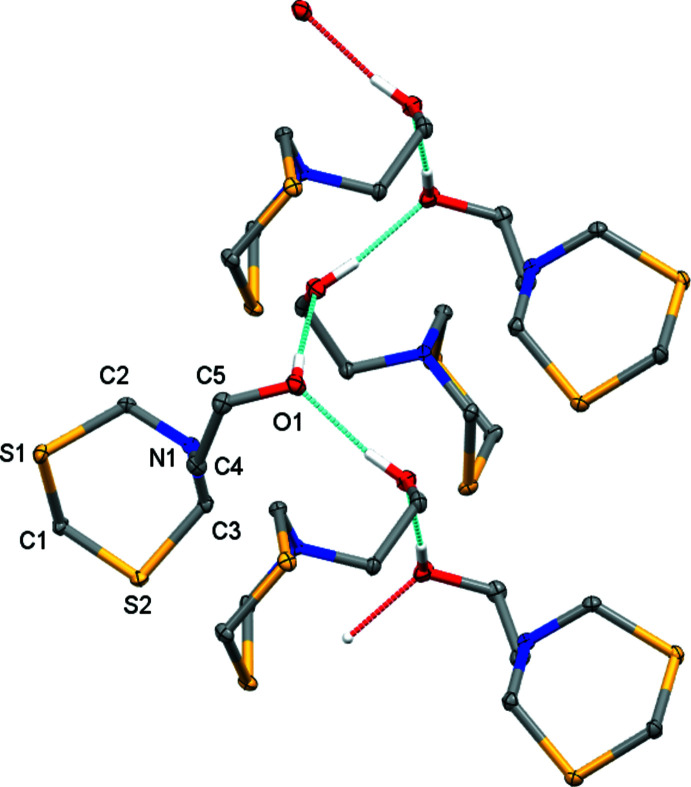
Hydrogen-bonding inter­actions in the form I polymorph. Mol­ecules are linked into infinite chains that extend along the *c*-axis direction. Hydrogen-bonded mol­ecules are symmetry-related to each other *via* the 4_1_-screw axis of the *I*4_1_/*a* space group. Coordinates from Wang *et al.* (2020 ▸). Displacement ellipsoids are shown at the 50% probability level. H atoms not involved in hydrogen-bonding are omitted for clarity.

**Figure 5:**
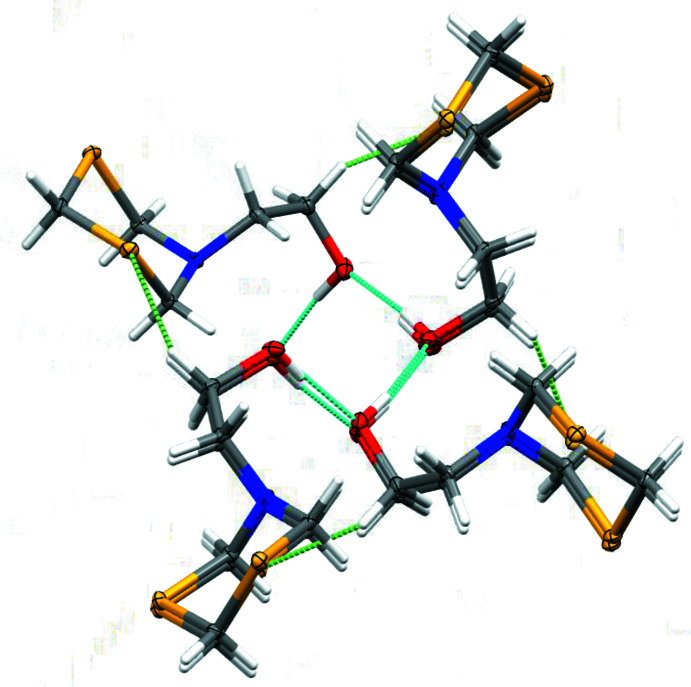
Partial packing view and hydrogen bonding in the form I polymorph, viewed at a slight angle along the *c* axis (4_1_-screw-axis direction). Turquoise dashed lines: O—H⋯O hydrogen bonds. Light-green dashed lines: weak C—H⋯S inter­actions. Coordinates from Wang *et al.* (2020 ▸). Displacement ellipsoids are shown at the 50% probability level.

Different inter­molecular inter­actions, especially hydrogen bonds, are the core reason for the formation of the two MEA-di­thia­zine polymorphs (form I and II). In the previously described crystal structure (form I), the mol­ecules are connected through O—H⋯O hydrogen bonds involving the hydroxyl O atoms as both hydrogen-bond donor and acceptor. A series of these inter­actions lead to the formation of one-dimensional, helical spirals in which adjacent mol­ecules are related to each other *via* the 4_1_ screw axis of the *I*4_1_/*a* space group (Figs. 4 ▸ and 5 ▸). The graph-set motif of the infinite strands is 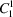 (2). The helical chains are further supported by weak C—H⋯S inter­actions involving the methyl­ene group adjacent to the OH group and one of the sulfur atoms of a mol­ecule related by three turns of the screw axis [C5⋯S2^i^ = 3.533 (3) Å; symmetry code: (i) = − 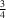 + *y*, 3/4 – *x*, 3/4 – *z*; Fig. 5 ▸]. No other significant inter­actions involving H atoms are observed, and individual helical spirals run parallel and anti-parallel to each other with no directional inter­actions between them. One other important aspect of the form-I polymorph is that the amine N atom does not act as a hydrogen-bond acceptor, neither towards the O—H group nor any of the methyl­ene CH_2_ hydrogen atoms.

**Figure 6:**
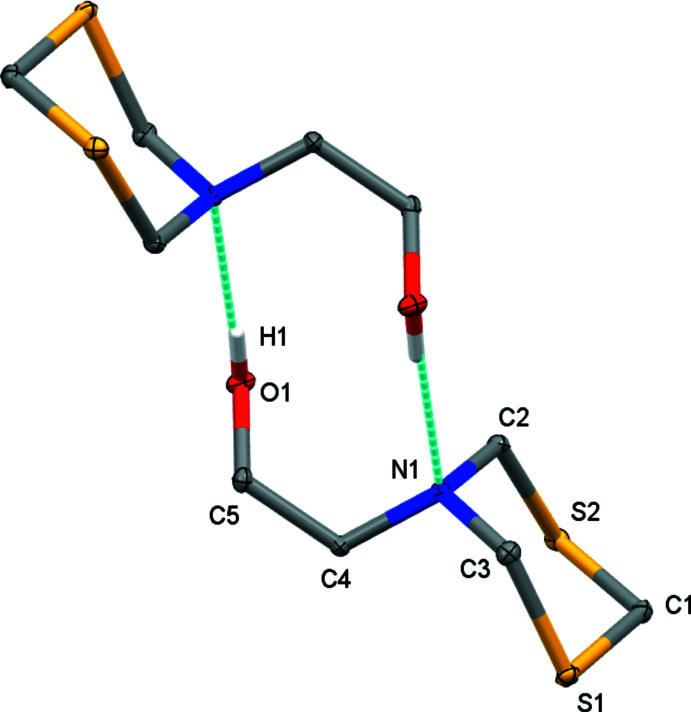
Hydrogen-bonding inter­actions in the form II polymorph. Mol­ecules are linked into centrosymmetric dimers connected *via* pairs of O—H⋯N hydrogen bonds. Displacement ellipsoids are shown at the 50% probability level.

In the form II structure described here, the amine N atom behaves differently and acts as the primary hydrogen-bond acceptor (Tables 2 ▸ and 3 ▸). Pairwise O—H⋯N [O⋯N = 2.9103 (8) Å] hydrogen bonds connect MEA-di­thia­zine mol­ecules into centrosymmetric dimers, with a graph-set notation of 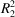 (10). (Table 3 ▸, Figs. 6 ▸ and 7 ▸). No O—H⋯O inter­actions are observed in the form II polymorph.

Contrary to the form I polymorph, where the spiral chains propagate in the *c*-axis direction with no significant directional inter­actions between parallel strands, the primary building units in the form II structure are connected to each other *via* C—H⋯O hydrogen bonds and weak C—H⋯S inter­actions. The C—H⋯O inter­actions are notably short for this kind of hydrogen bond [H⋯O = 2.42 Å, C⋯O = 3.3359 (9)Å]. The C—H⋯O inter­actions connect the hydrogen-bonded dimers into infinite layers lying perpendicular to (001).

The C—H⋯S inter­action is much weaker and connects parallel layers with each other. The layers, which are slightly corrugated, also inter­digitate with each other, yielding a densely packed and rigid three-dimensional arrangement. This is reflected in the density of the crystals in form II, which is (at 150 K) 1.468 g cm^−3^. The form I structure, with its lack of strong inter­actions between hydrogen-bonded spirals, is substanti­ally less densely packed (1.407 g cm^−3^ at 173 K, or 4.3% less dense than form II). This points towards form II likely being the thermodynamically more stable polymorph. This is also supported by the melting temperatures of the two forms, where form I has a reported melting onset of 314 K in comparison to 316 K measured for form II (Figure S1, supporting information). The difference in melting point between the two forms is small (∼2K); this could be coincidental or they might point towards a phase transformation of one of the two forms upon heating, with only one form being present once the melting point temperature is reached. For form II, a differential scanning calorimetry (DSC) investigation did show any indication of a phase change (Figure S2, supporting information). However, a thorough investigation of both polymorphs utilizing DSC would be necessary to determine the relative stabilities (Yu, 1995 ▸).

Crystals of the form I polymorph as reported by both Galvez-Ruiz *et al.* (2004 ▸) and Wang *et al.* (2020 ▸) are not the original material as isolated from the reaction of hydrogen sulfide with the MEA-triazine **1** scavenger in water as the solvent. Galvez-Ruiz *et al.* analyzed laboratory-prepared material that was obtained *via* a different route (reaction of ethano­lamine with NaHS and formaldehyde) and crystals were grown from chloro­form solution. In the 2020 report, the original material had been field samples obtained from an unspecified natural gas site, but the samples were purified and recrystallized prior to analysis. Field samples containing >90 wt% di­thia­zine were dissolved in isopropyl alcohol, filtered, and di­thia­zine crystals were obtained from iso­propanol–water mixtures by cooling to 278 K, and recrystallized twice to obtain large translucent crystals suitable for single-crystal structure analysis. All further analysis, including the measurement of the solubility of di­thia­zine in natural gas mixtures and variable pressure, was performed on the recrystallized samples. The original material from the field site was not further analyzed to verify that recrystallized and raw material were of the same kind, *i.e*., no powder XRD data of the original material were recorded, and no Rietveld analysis of the pattern was performed. The substantial difference in structure and density observed for the two polymorphs does indicate that they would also differ in other physicochemical properties. A noteworthy difference described herein is that crystalline material directly from the production facility and from laboratory recrystallization experiments were analyzed, and both resulted in the form II polymorph.

## Database survey

A search of the Cambridge Structural Database (CSD, version 5.42 of November 2020; Groom *et al.*, 2016 ▸) found a variety of hits featuring the 2-(1,3,5-di­thia­zinan-5-yl)ethanol structural backbone. The form I (*I*4_1_/*a*) polymorph of 2-(1,3,5-di­thia­zinan-5-yl)ethanol, collected at 223 K, was first reported in 2004 (CSD refcode ACAMIB; Galvez-Ruiz *et al.*, 2004 ▸) using room-temperature data, while a 173 K dataset of the same polymorph was reported in 2020 (ACAMIB01; Wang *et al.*, 2020 ▸). Form II, discussed in detail here, was recently disclosed as a CSD communication (ACAMIB02, Unruh 2021 ▸). When a phenyl group is added to the ethanol moiety, a discrete, monomeric unit results with the formation of an intra­molecular O—H⋯N (O⋯N = 2.782 Å) hydrogen bond (ACAMOH; Galvez-Ruiz *et al.*, 2004 ▸). Similarly, when a methyl group is appended (ACAMUN; Galvez-Ruiz *et al.*, 2004 ▸; ACAMUN01; Colorado-Peralta *et al.*, 2010 ▸), discrete monomers result with intra­molecular O—H⋯N (O⋯N = 2.721 and 2.732 Å) hydrogen bonds. However, when a phenyl and a methyl group are appended to the ethanol moiety (ACANAU; Galvez-Ruiz *et al.*, 2004 ▸), one-dimensional strands form through O—H⋯N (O⋯N = 3.145 Å) hydrogen bonds with a 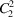 (10) graph-set motif. Three crystalline organic salts of 2-(1,3,5-di­thia­zinan-5-yl)ethanol have also been reported, including one with bromide and two with chloride counter-ions (HOSKIK and HOSKOQ; Bushmarinov *et al.*, 2009 ▸; HOSKOQ01; Galvez-Ruiz *et al.*, 2008 ▸). All three salts are isostructural, possessing a protonated nitro­gen atom and charge-assisted O—H⋯Br/Cl and charge-assisted N—H⋯Br/Cl hydrogen bonds and 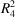 (14) graph sets. In all nine structures, the di­thia­zine ring adopts a chair conformation.

## Methods

Powder diffraction data were collected in focusing mode on a Panalytical Empyrean X-ray diffractometer equipped with Bragg–Brentano HD optics, a sealed-tube copper X-ray source (λ = 1.54178 Å), Soller slits on both the incident and receiving optics sides, and a PixCel3D Medipix detector. Samples were hand ground for 20 minutes using an agate mortar and pestle and packed in metal sample cups with a sample area 16 mm wide and 2 mm deep. 1/4° anti-scatter slits and 1/16° divergence slits as well as a 4 mm mask were chosen based on sample area and starting θ angle. Data were collected between 5 and 90° in 2θ using *Data Collector* software (PANalytical, 2019 ▸). Rietveld refinements were performed against the models of the single-crystal-structure data sets using the *HighScore* software (PANalytical, 2018 ▸). Refinement of preferred orientation was included using a spherical harmonics model.

Differential scanning calorimetry (DSC) and thermogravimetric analysis (TGA) data were measured using a TA Instruments Q20 DSC with heating rates of 5°C min^−1^. The sample was run in an alumina open pan from 25 to 100°C. The DSC and TGA traces are given in the Supporting Information (Fig. S3).

### Synthesis and crystallization

The commercially supplied MEA-triazine **1** solution, 47% active components in aqueous methanol (Innospec, Inc.), is stored in an external 650 gal polyethyl­ene tank and is dosed into two inclined static mixers with a piston-style positive displacement pump. A combined dose of 16 gal d^−1^ is administered to the two static mixers. Each mixer contains bead media that create a tortuous pathway that facilitates efficient mixing and provides the contact time for the hydrogen sulfide gases to react. One inclined static mixer services the well gases removed from the wellhead recovery line and the second services the excess gas from the stock-tank vapor-recovery system. The gaseous mixture of natural gas and hydrogen sulfide pass through the static mixer that contains the liquid MEA-triazine **1** solution. The MEA-triazine **1** solution is administered at a rate to keep the hydrogen sulfide below a targeted threshold and to ensure the di­thia­zine product **3** is transported through the static mixer at a sufficient rate to alleviate any plugging from reactionary solids.

The crystalline solids created through this process were collected and characterized by single-crystal and powder X-ray analysis as well as differential scanning calorimetry (DSC) and thermogravimetric analysis (TGA). The X-ray powder pattern was successfully indexed suggesting a single, pure bulk phase. Thermal analysis revealed negligible weight loss through 100°C by TGA, with a melt endotherm onset at 43°C (574 J g^−1^) and a peak temperature at 45°C.

Additionally, the crystalline material from the field equipment was recrystallized from aqueous iso­propanol. Approximately 100 mg of crystalline material was dissolved with gentle heating in approximately 10 ml of neat IPA or 50:50 IPA:water. The samples were allowed to cool to room temperature and slowly evaporate. After 24 h, a crystalline material resulted, which was filtered, dried and characterized by powder X-ray diffraction (Fig. S1, S2D–S2I, Tables S1B, S1C).

## Refinement

Single-crystal X-ray diffraction data, data collection and structure refinement details are summarized in Table 4 ▸. A common structural model was refined against the data collected at 150 K and at room temperature (no phase change was observed). The C-bound H atoms were positioned geometrically and constrained to ride on their parent atoms with C—H bond distances of 0.99 Å (at 150 K) and 0.97 Å (at room temperature). The positions of the hydroxyl H atoms were refined. *U*_iso_(H) values were set to 1.2*U*_eq_(C) or 1.5*U*_eq_(O).

## Supplementary Material

Crystal structure: contains datablock(s) 150K, RT, global. DOI: 10.1107/S2056989022000342/hb8010sup1.cif

Structure factors: contains datablock(s) 150K. DOI: 10.1107/S2056989022000342/hb8010150Ksup2.hkl

Structure factors: contains datablock(s) RT. DOI: 10.1107/S2056989022000342/hb8010RTsup3.hkl

CCDC references: 2134638, 2134637

Additional supporting information:  crystallographic information; 3D view; checkCIF report

## Figures and Tables

**Figure 7:**
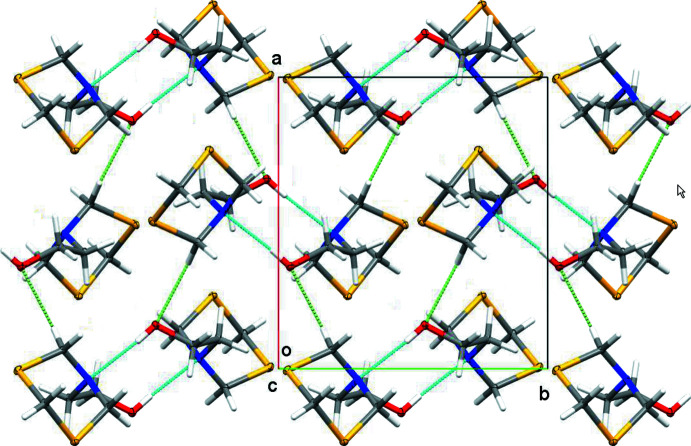
Partial packing view and hydrogen bonding in the form II polymorph, viewed down the *c* axis. Turquoise dashed lines: O—H⋯N hydrogen bonds. Light-green dashed lines: C—H⋯O inter­actions. Displacement ellipsoids are shown at the 50% probability level.

**Table 1:** Selected torsion angles (°) for the 150 K structure[Chem scheme1]

C2—S2—C1—S1	60.15 (4)	C4—N1—C3—S1	67.01 (7)
C3—S1—C1—S2	−60.52 (4)	C1—S1—C3—N1	60.35 (5)
C3—N1—C2—S2	65.52 (6)	C2—N1—C4—C5	−104.74 (6)
C4—N1—C2—S2	−68.05 (6)	C3—N1—C4—C5	122.32 (6)
C1—S2—C2—N1	−58.98 (5)	N1—C4—C5—O1	65.09 (7)
C2—N1—C3—S1	−66.53 (6)		

**Table 2:** Hydrogen-bond geometry (Å, °) for the 150 K[Chem scheme1] structure

*D*—H⋯*A*	*D*—H	H⋯*A*	*D*⋯*A*	*D*—H⋯*A*
O1—H1⋯N1^i^	0.792 (13)	2.124 (13)	2.9103 (8)	172.3 (13)
C3—H3*A*⋯O1^ii^	0.99	2.42	3.3359 (9)	154
C2—H2*A*⋯S1^iii^	0.99	3.01	3.9330 (7)	155
C3—H3*B*⋯S2^iv^	0.99	2.96	3.7899 (7)	142

Symmetry codes: (i) -x+1, -y, -z+1; (ii) x+{\script{1\over 2}}, -y+{\script{1\over 2}}, -z+1; (iii) -x+1, y-{\script{1\over 2}}, -z+{\script{3\over 2}}; (iv) x+{\script{1\over 2}}, y, -z+{\script{3\over 2}}.

**Table 3:** Hydrogen-bond geometry (Å, °) for the RT[Chem scheme1] structure

*D*—H⋯*A*	*D*—H	H⋯*A*	*D*⋯*A*	*D*—H⋯*A*
O1—H1⋯N1^i^	0.79 (2)	2.18 (2)	2.9627 (12)	171 (2)
C3—H3*A*⋯O1^ii^	0.97	2.50	3.3979 (14)	153
C2—H2*A*⋯S1^iii^	0.97	3.10	3.9985 (11)	156
C3—H3*B*⋯S2^iv^	0.97	3.05	3.8512 (11)	141

Symmetry codes: (i) -x+1, -y, -z+1; (ii) x+{\script{1\over 2}}, -y+{\script{1\over 2}}, -z+1; (iii) -x+1, y-{\script{1\over 2}}, -z+{\script{3\over 2}}; (iv) x+{\script{1\over 2}}, y, -z+{\script{3\over 2}}.

**Table 4:** Experimental details

	150 K	RT
Crystal data		
Chemical formula	C_5_H_11_NOS_2_	C_5_H_11_NOS_2_
*M*_r_	165.27	165.27
Crystal system, space group	Orthorhombic, *P* *b* *c* *a*	Orthorhombic, *P* *b* *c* *a*
Temperature (K)	150	296
*a*, *b*, *c* (Å)	9.5828 (4), 8.8467 (3), 17.6400 (8)	9.7160 (11), 8.9709 (10), 17.8577 (17)
*V* (Å^3^)	1495.45 (11)	1556.5 (3)
*Z*	8	8
Radiation type	Mo *K*α	Mo *K*α
μ (mm^−1^)	0.63	0.61
Crystal size (mm)	0.41 × 0.34 × 0.28	0.41 × 0.34 × 0.28

Data collection		
Diffractometer	Bruker AXS D8 Quest diffractometer with PhotonII charge-integrating pixel array detector (CPAD)	Bruker AXS D8 Quest diffractometer with PhotonII charge-integrating pixel array detector (CPAD)
Absorption correction	Multi-scan (*SADABS*; Krause *et al.*, 2015 ▸)	Multi-scan (*SADABS*; Krause *et al.*, 2015 ▸)
*T*_min_, *T*_max_	0.689, 0.747	0.696, 0.747
No. of measured, independent and observed [*I* > 2σ(*I*)] reflections	60184, 2857, 2609	47635, 2909, 2378
*R*_int_	0.040	0.040
(sin θ/λ)_max_ (Å^−1^)	0.770	0.765

Refinement		
*R*[*F* ^2^ > 2σ(*F* ^2^)], *wR*(*F* ^2^), *S*	0.018, 0.053, 1.06	0.027, 0.075, 1.02
No. of reflections	2857	2909
No. of parameters	86	86
H-atom treatment	H atoms treated by a mixture of independent and constrained refinement	H atoms treated by a mixture of independent and constrained refinement
Δρ_max_, Δρ_min_ (e Å^−3^)	0.38, −0.21	0.33, −0.27

Computer programs: *APEX3* and *SAINT* (Bruker, 2019 ▸), *SHELXS* (Sheldrick, 2008 ▸), *SHELXL2018/3* (Sheldrick, 2015 ▸), *shelXle* (Hübschle *et al.*, 2011 ▸), *Mercury* (Macrae *et al.*, 2020 ▸), and *publCIF* (Westrip, 2010 ▸).

## supplementary crystallographic information

### 2-(1,3,5-Dithiazinan-5-yl)ethanol (150K) Crystal data

**Table:**

C_5_H_11_NOS_2_	*D*_x_ = 1.468 Mg m^−^^3^
*M**_r_* = 165.27	Mo *K*α radiation, λ = 0.71073 Å
Orthorhombic, *P**b**c**a*	Cell parameters from 9803 reflections
*a* = 9.5828 (4) Å	θ = 2.3–33.1°
*b* = 8.8467 (3) Å	µ = 0.63 mm^−^^1^
*c* = 17.6400 (8) Å	*T* = 150 K
*V* = 1495.45 (11) Å^3^	Block, colourless
*Z* = 8	0.41 × 0.34 × 0.28 mm
*F*(000) = 704	

### 2-(1,3,5-Dithiazinan-5-yl)ethanol (150K) Data collection

**Table:**

Bruker AXS D8 Quest diffractometer with PhotonII charge-integrating pixel array detector (CPAD)	2857 independent reflections
Radiation source: fine focus sealed tube X-ray source	2609 reflections with *I* > 2σ(*I*)
Triumph curved graphite crystal monochromator	*R*_int_ = 0.040
Detector resolution: 7.4074 pixels mm^-1^	θ_max_ = 33.2°, θ_min_ = 3.1°
ω and phi scans	*h* = −14→14
Absorption correction: multi-scan (SADABS; Krause *et al.*, 2015)	*k* = −13→13
*T*_min_ = 0.689, *T*_max_ = 0.747	*l* = −27→27
60184 measured reflections	

### 2-(1,3,5-Dithiazinan-5-yl)ethanol (150K) Refinement

**Table:**

Refinement on *F*^2^	Secondary atom site location: difference Fourier map
Least-squares matrix: full	Hydrogen site location: mixed
*R*[*F*^2^ > 2σ(*F*^2^)] = 0.018	H atoms treated by a mixture of independent and constrained refinement
*wR*(*F*^2^) = 0.053	*w* = 1/[σ^2^(*F*_o_^2^) + (0.0247*P*)^2^ + 0.3304*P*] where *P* = (*F*_o_^2^ + 2*F*_c_^2^)/3
*S* = 1.06	(Δ/σ)_max_ = 0.002
2857 reflections	Δρ_max_ = 0.38 e Å^−^^3^
86 parameters	Δρ_min_ = −0.21 e Å^−^^3^
0 restraints	Extinction correction: SHELXL-2018/3 (Sheldrick, 2015), Fc^*^=kFc[1+0.001xFc^2^λ^3^/sin(2θ)]^-1/4^
Primary atom site location: structure-invariant direct methods	Extinction coefficient: 0.0043 (8)

### 2-(1,3,5-Dithiazinan-5-yl)ethanol (150K) Special details

**Table:**

Geometry. All esds (except the esd in the dihedral angle between two l.s. planes) are estimated using the full covariance matrix. The cell esds are taken into account individually in the estimation of esds in distances, angles and torsion angles; correlations between esds in cell parameters are only used when they are defined by crystal symmetry. An approximate (isotropic) treatment of cell esds is used for estimating esds involving l.s. planes.

### 2-(1,3,5-Dithiazinan-5-yl)ethanol (150K) Fractional atomic coordinates and isotropic or equivalent isotropic displacement parameters (Å^2^)

**Table:**

	*x*	*y*	*z*	*U*_iso_*/*U*_eq_	
S1	0.49529 (2)	0.46751 (2)	0.67098 (2)	0.01985 (5)	
S2	0.24939 (2)	0.25649 (2)	0.68835 (2)	0.01846 (5)	
O1	0.35020 (6)	0.04378 (6)	0.45260 (3)	0.02346 (11)	
H1	0.4068 (14)	−0.0193 (14)	0.4443 (7)	0.035*	
N1	0.46380 (6)	0.20571 (6)	0.58421 (3)	0.01538 (10)	
C1	0.38115 (7)	0.36827 (8)	0.73583 (4)	0.01980 (12)	
H1A	0.438074	0.301055	0.768347	0.024*	
H1B	0.334562	0.442853	0.769154	0.024*	
C2	0.36757 (7)	0.13153 (7)	0.63564 (4)	0.01697 (11)	
H2A	0.422566	0.072117	0.672647	0.020*	
H2B	0.310423	0.059314	0.606076	0.020*	
C3	0.56527 (7)	0.30023 (8)	0.62278 (4)	0.01882 (12)	
H3A	0.635243	0.334132	0.585154	0.023*	
H3B	0.614675	0.237470	0.660711	0.023*	
C4	0.39690 (8)	0.27738 (7)	0.51799 (4)	0.01898 (12)	
H4A	0.436105	0.379919	0.510816	0.023*	
H4B	0.295599	0.287903	0.527660	0.023*	
C5	0.41878 (7)	0.18614 (8)	0.44649 (4)	0.01854 (12)	
H5A	0.381227	0.242363	0.402461	0.022*	
H5B	0.519867	0.170101	0.438174	0.022*	

### 2-(1,3,5-Dithiazinan-5-yl)ethanol (150K) Atomic displacement parameters (Å^2^)

**Table:**

	*U*^11^	*U*^22^	*U*^33^	*U*^12^	*U*^13^	*U*^23^
S1	0.02283 (9)	0.01648 (8)	0.02024 (9)	−0.00517 (6)	0.00081 (6)	−0.00304 (5)
S2	0.01611 (8)	0.01798 (8)	0.02130 (9)	−0.00069 (5)	0.00337 (5)	0.00081 (5)
O1	0.0202 (2)	0.0179 (2)	0.0323 (3)	0.00111 (19)	−0.0025 (2)	−0.0054 (2)
N1	0.0183 (2)	0.0142 (2)	0.0136 (2)	0.00098 (18)	−0.00090 (18)	0.00015 (17)
C1	0.0236 (3)	0.0202 (3)	0.0156 (3)	−0.0008 (2)	0.0019 (2)	−0.0020 (2)
C2	0.0196 (3)	0.0126 (2)	0.0187 (3)	0.0001 (2)	0.0002 (2)	0.0012 (2)
C3	0.0150 (3)	0.0210 (3)	0.0204 (3)	−0.0004 (2)	0.0006 (2)	−0.0017 (2)
C4	0.0271 (3)	0.0151 (2)	0.0147 (3)	0.0028 (2)	−0.0025 (2)	0.0009 (2)
C5	0.0187 (3)	0.0225 (3)	0.0144 (3)	−0.0012 (2)	0.0011 (2)	−0.0004 (2)

### 2-(1,3,5-Dithiazinan-5-yl)ethanol (150K) Geometric parameters (Å, º)

**Table:**

S1—C1	1.8100 (7)	C1—H1B	0.9900
S1—C3	1.8338 (7)	C2—H2A	0.9900
S2—C1	1.8093 (7)	C2—H2B	0.9900
S2—C2	1.8355 (7)	C3—H3A	0.9900
O1—C5	1.4247 (9)	C3—H3B	0.9900
O1—H1	0.792 (13)	C4—C5	1.5120 (9)
N1—C2	1.4505 (8)	C4—H4A	0.9900
N1—C3	1.4517 (9)	C4—H4B	0.9900
N1—C4	1.4758 (8)	C5—H5A	0.9900
C1—H1A	0.9900	C5—H5B	0.9900
			
C1—S1—C3	97.04 (3)	N1—C3—S1	115.93 (5)
C1—S2—C2	97.65 (3)	N1—C3—H3A	108.3
C5—O1—H1	107.0 (9)	S1—C3—H3A	108.3
C2—N1—C3	113.16 (5)	N1—C3—H3B	108.3
C2—N1—C4	114.41 (5)	S1—C3—H3B	108.3
C3—N1—C4	114.49 (5)	H3A—C3—H3B	107.4
S2—C1—S1	113.22 (4)	N1—C4—C5	111.76 (5)
S2—C1—H1A	108.9	N1—C4—H4A	109.3
S1—C1—H1A	108.9	C5—C4—H4A	109.3
S2—C1—H1B	108.9	N1—C4—H4B	109.3
S1—C1—H1B	108.9	C5—C4—H4B	109.3
H1A—C1—H1B	107.7	H4A—C4—H4B	107.9
N1—C2—S2	115.88 (4)	O1—C5—C4	110.17 (5)
N1—C2—H2A	108.3	O1—C5—H5A	109.6
S2—C2—H2A	108.3	C4—C5—H5A	109.6
N1—C2—H2B	108.3	O1—C5—H5B	109.6
S2—C2—H2B	108.3	C4—C5—H5B	109.6
H2A—C2—H2B	107.4	H5A—C5—H5B	108.1
			
C2—S2—C1—S1	60.15 (4)	C4—N1—C3—S1	67.01 (7)
C3—S1—C1—S2	−60.52 (4)	C1—S1—C3—N1	60.35 (5)
C3—N1—C2—S2	65.52 (6)	C2—N1—C4—C5	−104.74 (6)
C4—N1—C2—S2	−68.05 (6)	C3—N1—C4—C5	122.32 (6)
C1—S2—C2—N1	−58.98 (5)	N1—C4—C5—O1	65.09 (7)
C2—N1—C3—S1	−66.53 (6)		

### 2-(1,3,5-Dithiazinan-5-yl)ethanol (150K) Hydrogen-bond geometry (Å, º)

**Table:**

*D*—H···*A*	*D*—H	H···*A*	*D*···*A*	*D*—H···*A*
O1—H1···N1^i^	0.792 (13)	2.124 (13)	2.9103 (8)	172.3 (13)
C3—H3*A*···O1^ii^	0.99	2.42	3.3359 (9)	154
C2—H2*A*···S1^iii^	0.99	3.01	3.9330 (7)	155
C3—H3*B*···S2^iv^	0.99	2.96	3.7899 (7)	142

Symmetry codes: (i) −*x*+1, −*y*, −*z*+1; (ii) *x*+1/2, −*y*+1/2, −*z*+1; (iii) −*x*+1, *y*−1/2, −*z*+3/2; (iv) *x*+1/2, *y*, −*z*+3/2.

### 2-(1,3,5-Dithiazinan-5-yl)ethanol (RT) Crystal data

**Table:**

C_5_H_11_NOS_2_	*D*_x_ = 1.411 Mg m^−^^3^
*M**_r_* = 165.27	Mo *K*α radiation, λ = 0.71073 Å
Orthorhombic, *P**b**c**a*	Cell parameters from 9354 reflections
*a* = 9.7160 (11) Å	θ = 3.1–32.9°
*b* = 8.9709 (10) Å	µ = 0.61 mm^−^^1^
*c* = 17.8577 (17) Å	*T* = 296 K
*V* = 1556.5 (3) Å^3^	Block, colourless
*Z* = 8	0.41 × 0.34 × 0.28 mm
*F*(000) = 704	

### 2-(1,3,5-Dithiazinan-5-yl)ethanol (RT) Data collection

**Table:**

Bruker AXS D8 Quest diffractometer with PhotonII charge-integrating pixel array detector (CPAD)	2909 independent reflections
Radiation source: fine focus sealed tube X-ray source	2378 reflections with *I* > 2σ(*I*)
Triumph curved graphite crystal monochromator	*R*_int_ = 0.040
Detector resolution: 7.4074 pixels mm^-1^	θ_max_ = 33.0°, θ_min_ = 3.3°
ω and phi scans	*h* = −14→14
Absorption correction: multi-scan (SADABS; Krause *et al.*, 2015)	*k* = −13→13
*T*_min_ = 0.696, *T*_max_ = 0.747	*l* = −27→27
47635 measured reflections	

### 2-(1,3,5-Dithiazinan-5-yl)ethanol (RT) Refinement

**Table:**

Refinement on *F*^2^	Secondary atom site location: difference Fourier map
Least-squares matrix: full	Hydrogen site location: mixed
*R*[*F*^2^ > 2σ(*F*^2^)] = 0.027	H atoms treated by a mixture of independent and constrained refinement
*wR*(*F*^2^) = 0.075	*w* = 1/[σ^2^(*F*_o_^2^) + (0.0313*P*)^2^ + 0.3838*P*] where *P* = (*F*_o_^2^ + 2*F*_c_^2^)/3
*S* = 1.02	(Δ/σ)_max_ = 0.001
2909 reflections	Δρ_max_ = 0.33 e Å^−^^3^
86 parameters	Δρ_min_ = −0.27 e Å^−^^3^
0 restraints	Extinction correction: SHELXL-2018/3 (Sheldrick, 2015), Fc^*^=kFc[1+0.001xFc^2^λ^3^/sin(2θ)]^-1/4^
Primary atom site location: isomorphous structure methods	Extinction coefficient: 0.030 (2)

### 2-(1,3,5-Dithiazinan-5-yl)ethanol (RT) Special details

**Table:**

Geometry. All esds (except the esd in the dihedral angle between two l.s. planes) are estimated using the full covariance matrix. The cell esds are taken into account individually in the estimation of esds in distances, angles and torsion angles; correlations between esds in cell parameters are only used when they are defined by crystal symmetry. An approximate (isotropic) treatment of cell esds is used for estimating esds involving l.s. planes.
Refinement. solved by isomorphous replacement from its 150 K structure

### 2-(1,3,5-Dithiazinan-5-yl)ethanol (RT) Fractional atomic coordinates and isotropic or equivalent isotropic displacement parameters (Å^2^)

**Table:**

	*x*	*y*	*z*	*U*_iso_*/*U*_eq_	
S1	0.49595 (3)	0.46191 (3)	0.67128 (2)	0.04261 (9)	
S2	0.25087 (3)	0.25541 (3)	0.68813 (2)	0.03921 (8)	
O1	0.35211 (10)	0.04855 (10)	0.45103 (6)	0.0515 (2)	
H1	0.408 (2)	−0.014 (2)	0.4441 (10)	0.077*	
N1	0.46246 (8)	0.20389 (9)	0.58454 (4)	0.03187 (16)	
C1	0.38192 (12)	0.36489 (12)	0.73521 (5)	0.0410 (2)	
H1A	0.337314	0.437407	0.767376	0.049*	
H1B	0.436245	0.299490	0.766794	0.049*	
C2	0.36706 (11)	0.13141 (10)	0.63551 (5)	0.03458 (19)	
H2A	0.419986	0.073459	0.671202	0.042*	
H2B	0.311291	0.062112	0.606907	0.042*	
C3	0.56354 (10)	0.29615 (12)	0.62292 (6)	0.0390 (2)	
H3A	0.631288	0.328685	0.586523	0.047*	
H3B	0.610954	0.234689	0.659408	0.047*	
C4	0.39676 (13)	0.27614 (11)	0.51883 (5)	0.0400 (2)	
H4A	0.433847	0.375837	0.512923	0.048*	
H4B	0.298622	0.285012	0.527739	0.048*	
C5	0.42022 (11)	0.18902 (13)	0.44745 (5)	0.0401 (2)	
H5A	0.385530	0.245413	0.405120	0.048*	
H5B	0.518119	0.173540	0.440156	0.048*	

### 2-(1,3,5-Dithiazinan-5-yl)ethanol (RT) Atomic displacement parameters (Å^2^)

**Table:**

	*U*^11^	*U*^22^	*U*^33^	*U*^12^	*U*^13^	*U*^23^
S1	0.04861 (16)	0.03562 (14)	0.04361 (15)	−0.01122 (11)	0.00100 (11)	−0.00706 (10)
S2	0.03421 (13)	0.03855 (14)	0.04485 (15)	−0.00120 (9)	0.00745 (10)	0.00121 (10)
O1	0.0443 (4)	0.0425 (4)	0.0676 (6)	0.0036 (4)	−0.0086 (4)	−0.0147 (4)
N1	0.0372 (4)	0.0300 (3)	0.0284 (3)	0.0038 (3)	−0.0015 (3)	−0.0002 (3)
C1	0.0503 (6)	0.0417 (5)	0.0311 (4)	−0.0001 (4)	0.0031 (4)	−0.0045 (4)
C2	0.0406 (5)	0.0254 (4)	0.0377 (4)	0.0000 (3)	−0.0008 (4)	0.0022 (3)
C3	0.0306 (4)	0.0447 (5)	0.0416 (5)	−0.0010 (4)	0.0017 (4)	−0.0030 (4)
C4	0.0572 (6)	0.0324 (4)	0.0303 (4)	0.0064 (4)	−0.0054 (4)	0.0014 (3)
C5	0.0394 (5)	0.0518 (6)	0.0291 (4)	−0.0008 (4)	0.0026 (4)	−0.0003 (4)

### 2-(1,3,5-Dithiazinan-5-yl)ethanol (RT) Geometric parameters (Å, º)

**Table:**

S1—C1	1.8134 (11)	C1—H1B	0.9700
S1—C3	1.8407 (11)	C2—H2A	0.9700
S2—C1	1.8145 (11)	C2—H2B	0.9700
S2—C2	1.8425 (10)	C3—H3A	0.9700
O1—C5	1.4248 (15)	C3—H3B	0.9700
O1—H1	0.79 (2)	C4—C5	1.5125 (14)
N1—C2	1.4527 (13)	C4—H4A	0.9700
N1—C3	1.4558 (13)	C4—H4B	0.9700
N1—C4	1.4847 (12)	C5—H5A	0.9700
C1—H1A	0.9700	C5—H5B	0.9700
			
C1—S1—C3	97.22 (5)	N1—C3—S1	116.07 (7)
C1—S2—C2	97.66 (5)	N1—C3—H3A	108.3
C5—O1—H1	107.2 (15)	S1—C3—H3A	108.3
C2—N1—C3	112.95 (8)	N1—C3—H3B	108.3
C2—N1—C4	114.60 (8)	S1—C3—H3B	108.3
C3—N1—C4	114.45 (8)	H3A—C3—H3B	107.4
S1—C1—S2	113.38 (5)	N1—C4—C5	112.07 (8)
S1—C1—H1A	108.9	N1—C4—H4A	109.2
S2—C1—H1A	108.9	C5—C4—H4A	109.2
S1—C1—H1B	108.9	N1—C4—H4B	109.2
S2—C1—H1B	108.9	C5—C4—H4B	109.2
H1A—C1—H1B	107.7	H4A—C4—H4B	107.9
N1—C2—S2	116.12 (6)	O1—C5—C4	110.43 (9)
N1—C2—H2A	108.3	O1—C5—H5A	109.6
S2—C2—H2A	108.3	C4—C5—H5A	109.6
N1—C2—H2B	108.3	O1—C5—H5B	109.6
S2—C2—H2B	108.3	C4—C5—H5B	109.6
H2A—C2—H2B	107.4	H5A—C5—H5B	108.1
			
C3—S1—C1—S2	−60.08 (7)	C4—N1—C3—S1	67.18 (10)
C2—S2—C1—S1	59.74 (7)	C1—S1—C3—N1	60.12 (9)
C3—N1—C2—S2	65.59 (10)	C2—N1—C4—C5	−106.30 (10)
C4—N1—C2—S2	−67.90 (9)	C3—N1—C4—C5	120.91 (10)
C1—S2—C2—N1	−59.00 (8)	N1—C4—C5—O1	66.01 (12)
C2—N1—C3—S1	−66.38 (10)		

### 2-(1,3,5-Dithiazinan-5-yl)ethanol (RT) Hydrogen-bond geometry (Å, º)

**Table:**

*D*—H···*A*	*D*—H	H···*A*	*D*···*A*	*D*—H···*A*
O1—H1···N1^i^	0.79 (2)	2.18 (2)	2.9627 (12)	171 (2)
C3—H3*A*···O1^ii^	0.97	2.50	3.3979 (14)	153
C2—H2*A*···S1^iii^	0.97	3.10	3.9985 (11)	156
C3—H3*B*···S2^iv^	0.97	3.05	3.8512 (11)	141

Symmetry codes: (i) −*x*+1, −*y*, −*z*+1; (ii) *x*+1/2, −*y*+1/2, −*z*+1; (iii) −*x*+1, *y*−1/2, −*z*+3/2; (iv) *x*+1/2, *y*, −*z*+3/2.
